# Inhibition of complement C5a receptor protects lung cells and tissues against lipopolysaccharide-induced injury via blocking pyroptosis

**DOI:** 10.18632/aging.202671

**Published:** 2021-03-10

**Authors:** Renying Wang, Yunxing Wang, Lan Hu, Zhenbing Lu, Xiaoshan Wang

**Affiliations:** 1 Department of Emergency, Ruijin Hospital, Shanghai Jiao Tong University, School of Medicine, Shanghai 201801, China

**Keywords:** acute lung injury, complement, C5a, inflammation, pyroptosis

## Abstract

Acute lung injury (ALI) is the injury of alveolar epithelial cells and capillary endothelial cells caused by various factors. Complement system and pyroptosis have been proved to be involved in ALI, and inhibition of C5a/C5a receptor (C5aR) could alleviate ALI. This study aimed to investigate whether C5a/C5aR inhibition could protect against LPS-induced ALI via mediating pyroptosis. Rats were assigned into four groups: Control, LPS, LPS+W-54011 1mg/kg, and LPS+W-54011 5mg/kg. Beas-2B cells pretreated with or without C5a and W-54011, alone and in combination, were challenged with LPS+ATP. Results unveiled that LPS caused lung tissue injury and inflammatory response, increased pyroptotic and apoptotic factors, along with elevated C5a concentration and C5aR expressions. However, W-54011 pretreatment alleviated lung damage and pulmonary edema, reduced inflammation and prevented cell pyroptosis. *In vitro* studies confirmed that LPS+ATP reduced cell viability, promoted cell death, generated inflammatory factors and promoted expressions of pyroptosis-related proteins, which could be prevented by W-54011 pretreatment while intensified by C5a pretreatment. The co-treatment of C5a and W-54011 could blunt the effects of C5a on LPS+ATP-induced cytotoxicity. In conclusion, inhibition of C5a/C5aR developed protective effects against LPS-induced ALI and the cytotoxicity of Beas-2B cells, and these effects may depend on blocking pyroptosis.

## INTRODUCTION

Sepsis is a systemic inflammatory response syndrome owing to clear or suspected infections, and it can progress into severe sepsis and septic shock, both of which are crucial clinical challenges in intensive care medicine. Millions are diagnosed with sepsis every year, responsible for one fourth of mortality [1]. Although great progress has been made in reducing mortality over the past few years, sepsis remains one of the primary causes of death among patients in intensive care units [2, 3]. Among the manifestations, organ dysfunction is the most serious one, which can lead to critical chronic illnesses. The lungs are the most vulnerable organs in response to the onset of sepsis that can cause acute lung injury (ALI) [4]. ALI is featured by the damage of alveolar epithelial cells and capillary endothelial cells, which results in alveolar edema, insufficient lung function, uncontrolled ventilation/blood flow ratio, and even the emergence of respiratory distress syndrome [5].

The immune system and immunity-induced inflammation widely partake in the pathogenesis of sepsis and ALI [6]. Therefore, sepsis has traditionally been recognized as a result of an uncontrolled inflammatory response that provokes organ dysfunction [7]. Complement system, mainly composed of C3, C4 and C5 components, is the central component of immune system. Complement system can serve as the first line of defense against pathogens and stressed host cells by interacting with each other to handle pathogens and trigger inflammatory responses [8]. Complement system has been widely identified as a facilitator in the development of sepsis and sepsis-induced ALI. For example, the anaphylatoxins C5a, produced by the complement C5, is the most effective inflammatory peptide and it is strongly activated during sepsis. C5a is indirectly involved in apoptosis, leading to destruction of the central immune system [9]. The activation of C5a complement could trigger release of products of nucleosomes (such as histones), resulting in a circle of tissue injury and inflammation during ALI [10]. The blockade of C5a or its receptor C5aR inhibits the development of sepsis and significantly improves the survival rate of animal models [11, 12]. Therefore, C5a is a pivotal factor for the pathogenesis of sepsis.

Besides, *in vivo* results from a recent paper have demonstrated that C5a could enhance inflammasome NLRP3 (nucleotide-binding oligomerization domain-like receptor with pyrin domain) and Caspase1 activation, thereby augmenting the release of pro-inflammatory factors IL-1β and IL-18 during septic cardiomyopathy [13]. The inflammasome NLRP3 is a multi-protein complex that mediates the stimulation of proteins associated with the maturation, secretion of pro-inflammatory cytokines, IL-1β and IL-18 contained, and induction of pyroptosis. Pyroptosis is a pattern of programmed cell death, consisting of canonical and non-canonical pathways. In non-canonical pathway, exogenous cytotoxins like lipopolysaccharide (LPS) can directly facilitate the activation of Caspase 11, Caspase 4, Caspase 5 and result in the cleavage of pyroptosis executor gasdermin D (GSDMD) and perforation of the cell membrane. Further, the N-terminal of GSDMD (GSDMD-N) activates NLRP3, thus evoking the release of IL-1β and IL-18. In the canonical pathway, activated NLRP3 could stimulate Caspase 1, and lead to cleavage of GSDMD as well as generation of IL-1β and IL-18. Besides, high mobility group box 1 (HMGB1) is also released into the extracellular matrix during pyroptosis, causing activation and amplification of inflammation and immunity. Importantly, pyroptosis has been confirmed to play a crucial role in the development of sepsis and sepsis-affected organ dysfunction, exerting potent pro-inflammatory actions [14–16]. Moreover, the blockade of NLRP3 or Caspase 1 can remarkably protect mice model against LPS-induced acute lung injury [17]. In addition, the inhibition of pyroptosis also displays protective effects against lung injury [18, 19]. However, whether C5a could mediate cell pyroptosis to ultimately affect the development of sepsis-induced ALI remains unknown.

In this study, considering the crucial roles of complement C5a and pyroptosis in inflammation and sepsis, we speculated that blockade of C5a or C5aR could alleviate inflammatory response and pyroptosis, thus protecting lung cells and tissues against LPS-induced injury. C5aR inhibitor W-54011 [20] was utilized to verify our hypothesis *in vivo* and *in vitro*.

## RESULTS

### W-54011 alleviates LPS-induced lung injury

**Figure 1:**
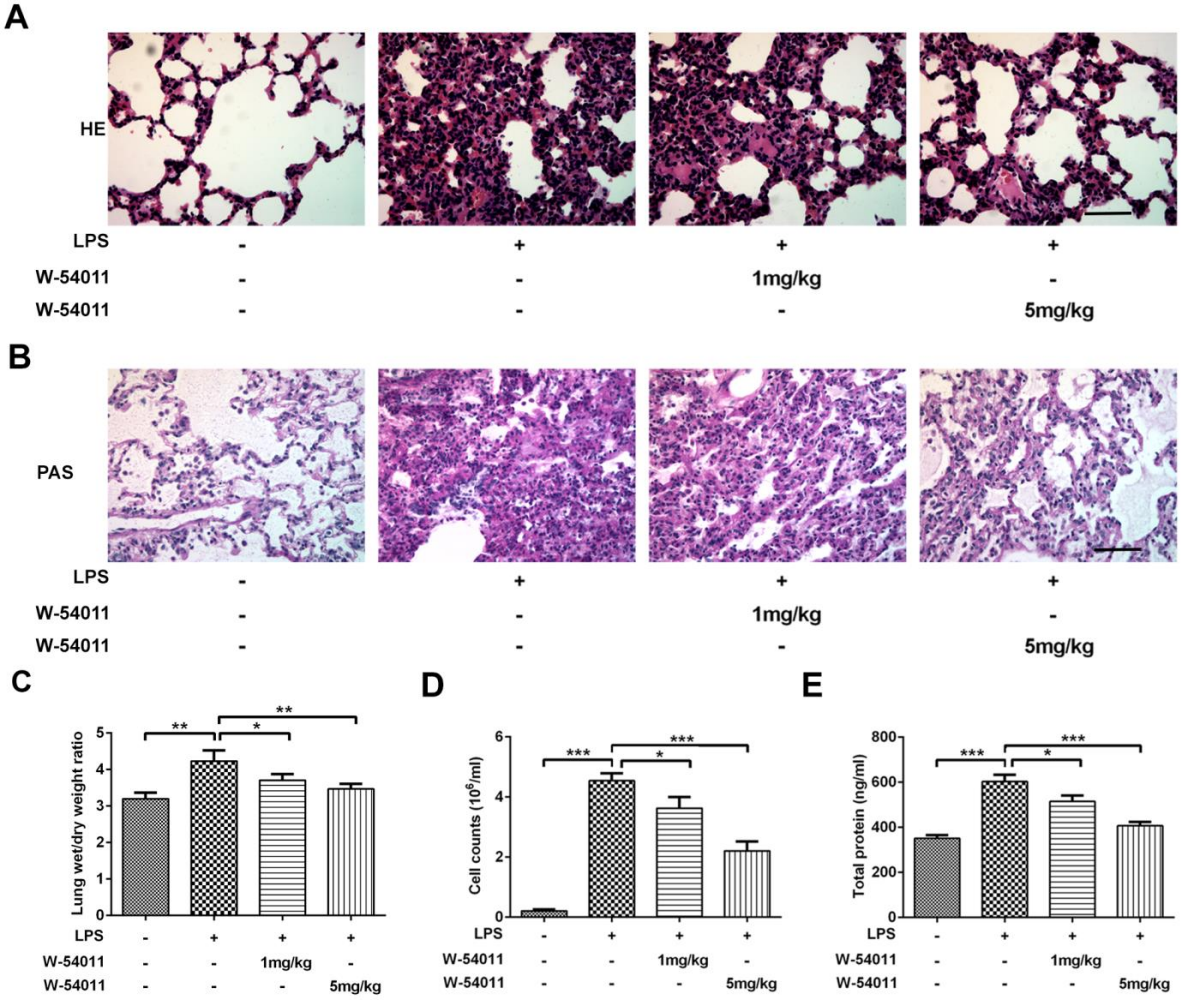
**Alterations of histological evaluation, lung wet/dry ratio, BALF cell counts and total protein level.** (**A**) Morphological changes in the lung by H&E staining. The sections were stained with hematoxylin (blue, nucleus) and eosin (pink, muscle fiber). The scale bar represents 50 μm. (**B**) Morphological changes in the lung by Periodic Acid Schiff (PAS) staining. The sections were stained with hematoxylin (blue, nucleus) and PAS (pink, glycogen). The scale bar represents 50 μm. (**C**–**E**) Effect of W-54011 on lung wet/dry ratio (**C**), BALF cell counts (**D**) and total protein level (**E**) 12 h after intratracheal instillation of LPS. n = 7. ^*^P < 0.05, ^**^P < 0.01 and ^***^P < 0.001.

To investigate the effects of C5a/C5aR inhibition on ALI, we employed an LPS-induced lung injury rat model and applied the C5aR inhibitor W-54011 with doses of 1mg/kg and 5mg/kg. The histological characteristics and the degree of lung injury in the LPS-induced ALI rat model were discovered, as exhibited in Figure 1A, 1B. Compared to the lung tissues of control rats, those of rats undergoing LPS administration showed obvious pathological alterations including intra-alveolar hemorrhage, inter-alveolar septum thickening, pulmonary interstitial edema and inflammatory cells infiltration, indicating the occurrence of ALI. However, with both 1mg/kg and 5mg/kg W-54011 pretreatment, the lung structure nearly returned to the normal appearance and the inflammatory cells infiltration was improved. Meanwhile, rats subjected to LPS displayed elevated Wet/Dry ratios compared to the control rats, which were significantly reduced by W-54011 pretreatment (Figure 1C). These data demonstrated the protective effect of C5aR inhibition on LPS-induced lung damage.

The BALF of animals was also collected to examine LPS-induced lung injury. LPS triggered a significant increase of cell amount in BALF, whereas W-54011 pretreatment dramatically decreased the count (Figure 1D). In addition, the total protein concentration of BALF can be applied to assess pulmonary capillary permeability, the increase of which could lead to pulmonary edema [21]. Compared with the control group, the total protein concentration of BALF in the LPS group was much higher (Figure 1E). Nevertheless, W-54011 pretreatment reversed the promotion of LPS on total protein concentration of BALF, suggesting that the inhibition of complement C5a/C5aR by W-54011 prevented higher capillary permeability after LPS stimulation (Figure 1E).

### W-54011 significantly decreases C5aR influx, levels of inflammatory factors, and cell pyroptosis in LPS-challenged rats

**Figure 2:**
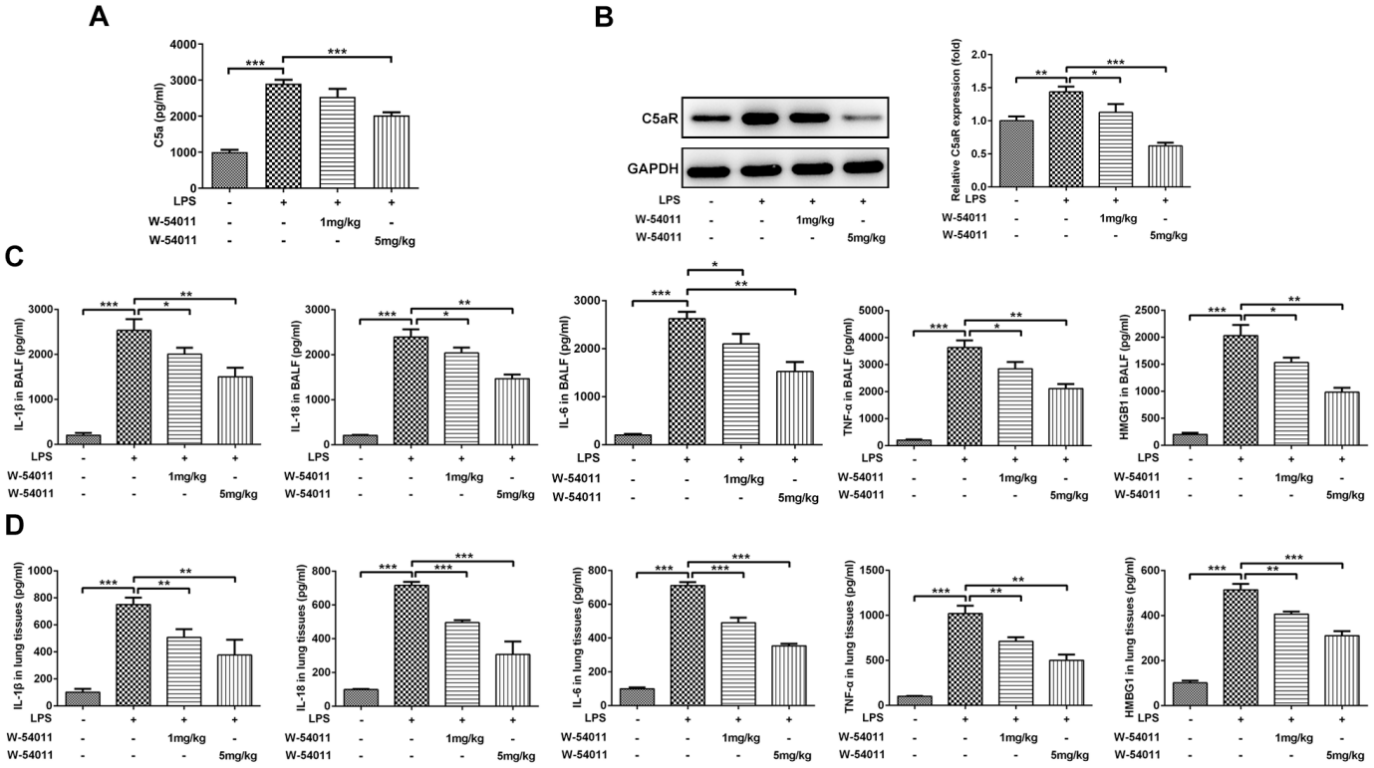
**Alterations of C5aR and proinflammatory cytokines level.** (**A**) The content of C5a in serum was measured by ELISA assay. (**B**) The protein expression of C5aR in lung tissues were detected by western blotting. (**C**, **D**) The concentrations of pro-inflammatory cytokines including IL-1β, IL-18, IL-6, TNF-α and HMGB1 in BALF (**C**) and the lung tissues (**D**) were measured by ELISA kits, respectively. n = 7. ^*^P < 0.05, ^**^P < 0.01 and ^***^P < 0.001.

To test the inhibitory efficacy of W-54011 on C5a/C5aR, the content of C5a in serum and level of C5aR in lung tissues were detected. As shown in Figure 2A, 2B, LPS stimulation increased the level of C5a in serum and C5aR in lung tissues. Whereas, W-54011 slightly reduced C5a content and significantly decreased C5aR level, compared with the LPS group. These results implied the potent suppressive effect of W-54011 on the interaction between C5a and its receptor C5aR.

The concentrations of pro-inflammatory cytokines including IL-1β, IL-18, TNF-α, IL-6 and HMGB1 in BALF and lung tissues were presented in Figure 2C, 2D. Results presented that the expressions of IL-1β, IL-18, TNF-α, IL-6 and HMGB1 were distinctly increased in LPS-induced ALI. However, W-54011 pretreatment suppressed the elevated expressions of these pro-inflammatory cytokines in both the BALF and the lung tissues.

**Figure 3:**
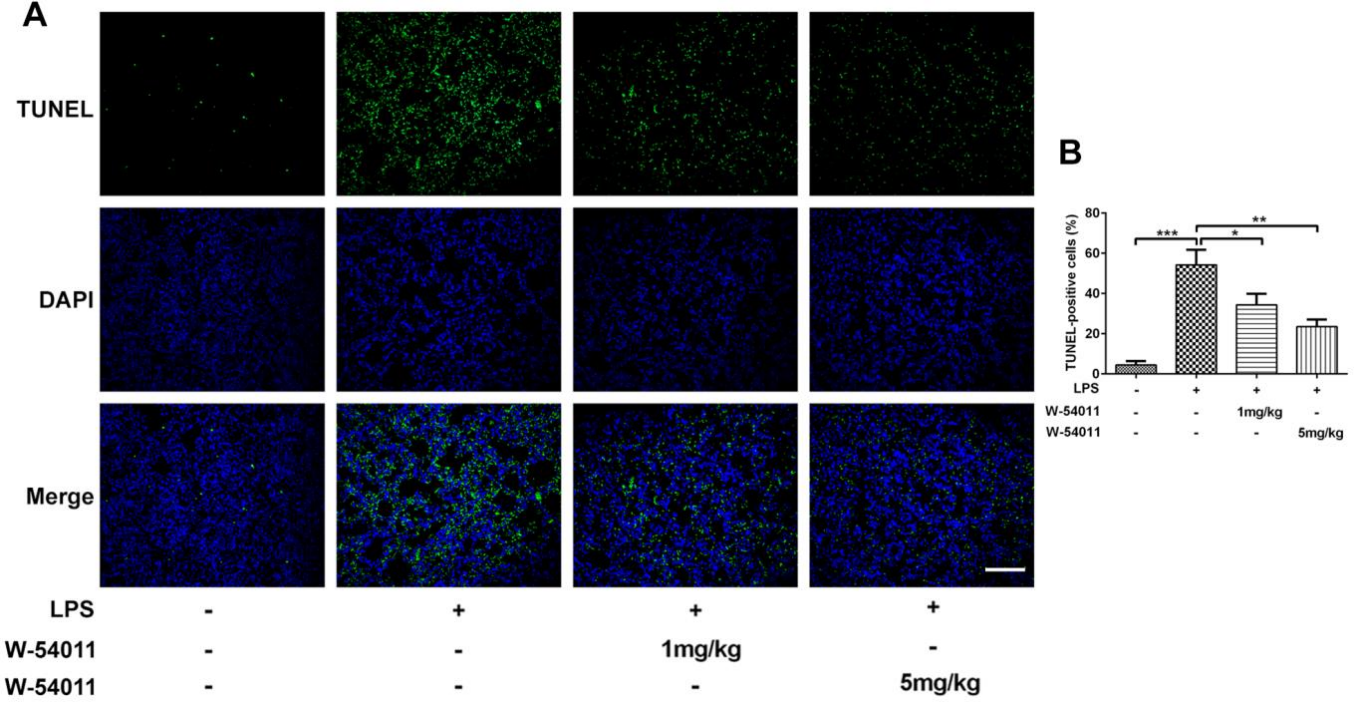
**The effect of W-54011 on TUNEL-positive cells.** (**A**) Representative TUNEL staining for lung tissues. Dead cells were stained with green and DAPI was used for staining the nucleus (blue). The scale bar represents 50 μm. (**B**) Quantitative analysis for the number of TUNEL-positive cells in different groups (n = 7). ^*^P < 0.05, ^**^P < 0.01 and ^***^P < 0.001.

**Figure 4:**
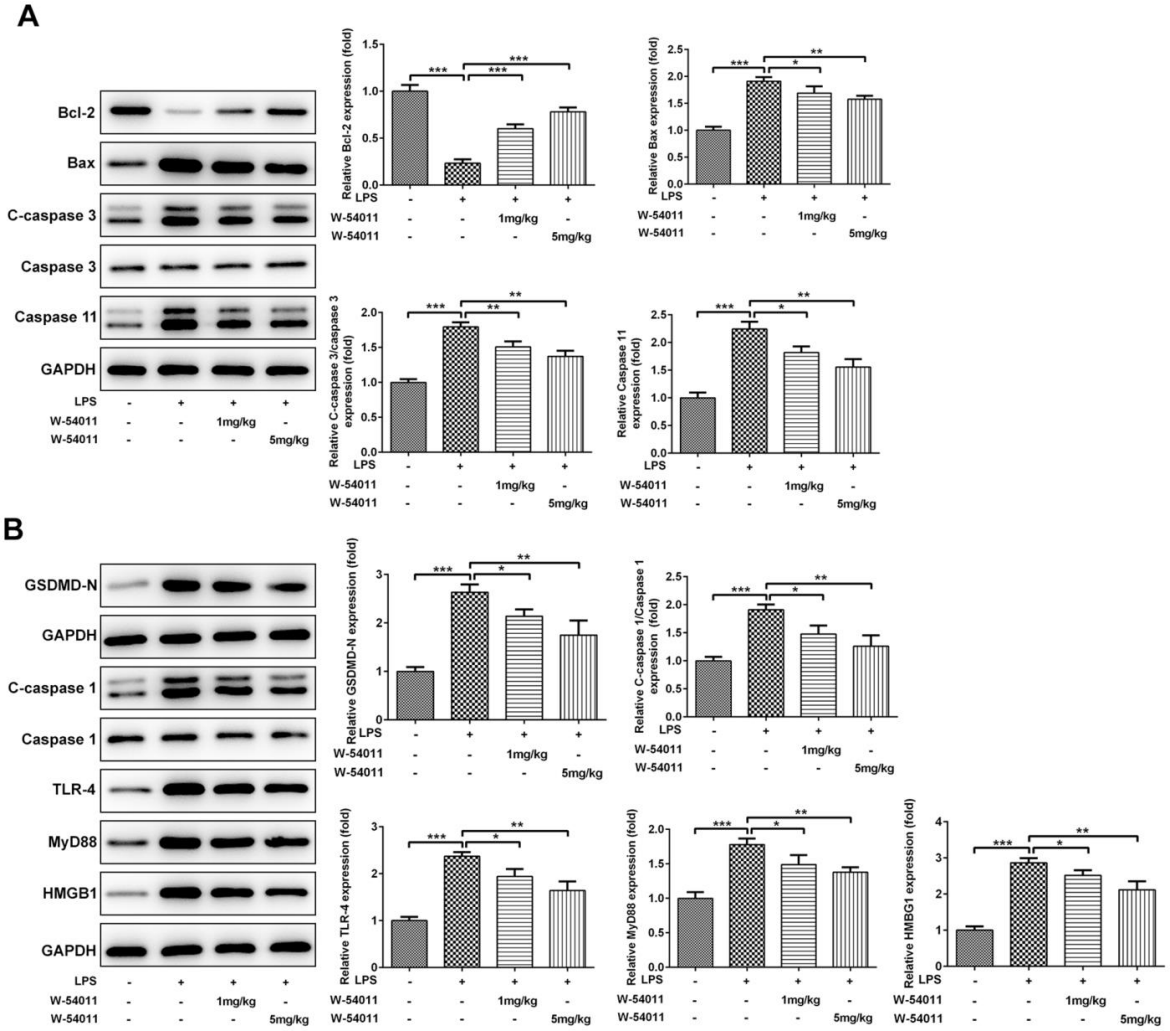
**The effect of W-54011 on the expression of proteins related to apoptosis and pyroptosis in lung tissues.** (**A**) The expression of apoptosis-related proteins including Bcl-2, Bax, Caspase 3 and Caspase 11 in different groups was detected using western blotting. (**B**) The expression of pyroptosis-related proteins including GSDMD, Caspase 1, TLR-4, MyD88 and HMGB1 in different groups was dissected using western blotting. The bar graphs represent the mean gray value of the protein bands after normalized to GAPDH. All data was achieved from 3 times independent replicated experiments. n = 7. ^*^P < 0.05, ^**^P < 0.01 and ^***^P < 0.001.

TUNEL staining was utilized to detect cell death of lung tissues. Results from Figure 3 revealed that LPS-induced ALI led to an increase in DNA fragmentation, but this change was alleviated by W-54011 addition. Next, the expressions of proteins involved in apoptosis and pyroptosis were tested via western blot. As displayed in Figure 4, the expressions of Bax, cleaved-Caspase 3/11, GSDMD-N, cleaved-Caspase 1, HMGB1, TLR4 and its downstream binding molecule MyD88 were up-regulated, while Bcl-2 was down-regulated in lung tissues after LPS challenge, hinting the activation of apoptosis, pyroptosis and TLR4/MyD88-mediated inflammation upon the stimulation of LPS. Moreover, W-54011 significantly rescued the enhancement of GSDMD-N, cleaved-Caspase 1, HMGB1, TLR4 and MyD88, together with Bax, Bcl-2, and cleaved-Caspase 3/11, compared with LPS group. The results together indicated that the inhibition of C5a/C5aR exerted remarkably effects on apoptosis and pyroptosis pathway.

### W-54011 recovers cell viability, as well as prevents inflammatory response and cell pyroptosis in LPS+ATP-induced cells in the presence of C5a

**Figure 5:**
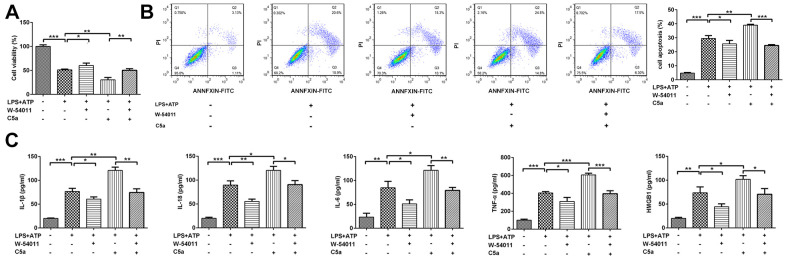
**The effect of W-54011 on cell viability and pro-inflammatory cytokines levels in the presence of C5a in Beas-2B cells.** (**A**) The cell viability in different groups was detected by CCK-8 assay. (**B**) Representative flow cytometry and quantitative analyses for dead cells. (**C**) The concentrations of pro-inflammatory cytokines including IL-1β, IL-18, IL-6, TNF-α and HMGB1 in supernatant of Beas-2B cells were determined by ELISA assay. Each experiment was repeated at least 3 times. ^*^P < 0.05, ^**^P < 0.01 and ^***^P < 0.001.

To further confirm our *in vivo* findings and the possible mechanism involved in regulation of W-54011, Beas-2B cells were treated with W-54011, C5a or C5a+W-54011 prior to LPS+ATP stimulation. Alterations of cell viability, inflammatory cytokines, apoptosis and pyroptosis-related proteins were observed in cell experiment. In contrast to control cells, LPS+ATP resulted in decreased cell viability, increased cell death and induced inflammatory factors, which were abrogated by W-54011 treatment. Opposite results of C5a treatment were found in contrast with W-54011 treatment in LPS+ATP-treated cells. Besides, the co-treatment of W-54011 could neutralize the actions of C5a (Figure 5).

**Figure 6:**
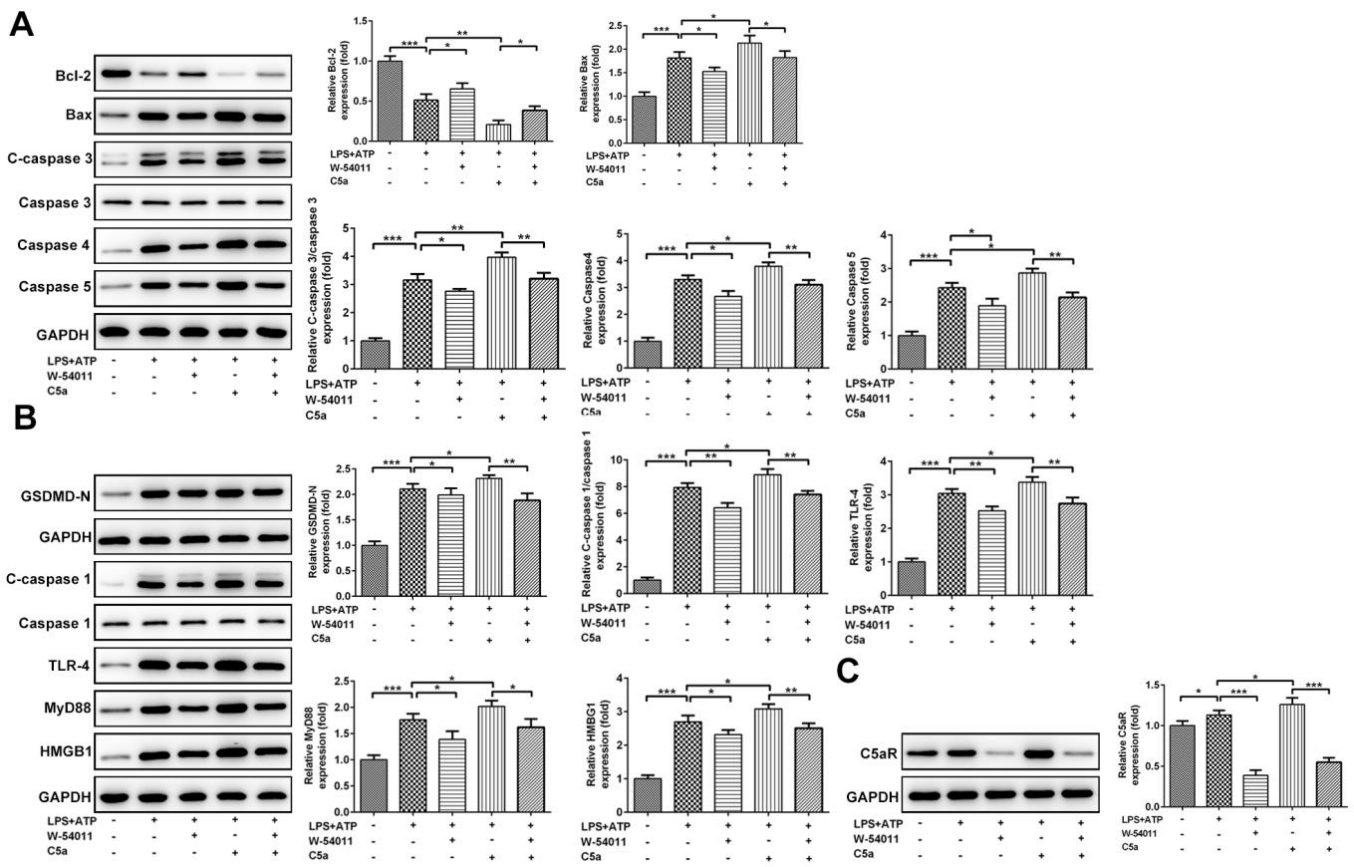
**The effect of W-54011 on the expression of proteins related to apoptosis and pyroptosis in Beas-2B cells.** (**A**) The expression of apoptosis-related proteins including Bcl-2, Bax, Caspase 3, Caspase 4 and Caspase 5 in different groups was detected using western blotting. (**B**) The expression of pyroptosis-related proteins including GSDMD, Caspase 1, TLR-4, MyD88 and HMGB1 in different groups was analyzed using western blotting. (**C**) The protein expression of C5aR in Beas-2B cells was analyzed using western blotting. Each experiment was repeated at least 3 times. The bar graphs represent the mean gray value of the protein bands after normalized to GAPDH. All data was achieved from 3 times independent replicated experiments. ^*^P < 0.05, ^**^P < 0.01 and ^***^P < 0.001.

Results from Figure 6 demonstrated that C5aR level and the expressions of proteins associated with apoptosis and pyroptosis, including Bax, cleaved-Caspase 3/4/5, GSDMD-N, cleaved-Caspase 1, HMGB1, TLR4 and MyD88, were all increased by treatment of LPS+ATP. However, the expressions of these proteins induced by LPS+ATP were weakened in the presence of W-54011 but strengthened by C5a. And the co-treatment of W-54011 also blunted the effects of C5a.

## DISCUSSION

The complement system has been illustrated in the pathogenesis of various inflammatory diseases. It functions through the generation of anaphylatoxins (C3a, C4a, and C5a) by the cleavage of the C3, C4, and C5 components. Considering the participation of C3, C4, and C5 in inflammation, complement inhibitors are introduced as drug candidates for the treatment of complement-dependent inflammatory diseases [22].

Although the complement system is a promising target for therapeutic intervention, there are few works mentioning this possibility. Undoubtedly, the availability of the first clinical complement drug, anti-C5 antibody eculizumab (Soliris®, Alexion Pharmaceuticals), has helped us gain clinical experience and expand the scope of indications [23]. In 2018, Herrmann et al [12] reported that the specific genetic or pharmacological disruption of C5aR1 can rapidly ameliorate the symptoms of septic animals by suppressing the pathogenic inflammatory response. To the best of our knowledge, the present work is the first study utilizing a C5aR inhibitor to control LPS-induced lung injury.

It has been established that the LPS from Gram-negative bacteria provokes immune response and inflammatory reaction via activating multiple molecular pathways. Above all, TLR4, as a mammalian receptor for LPS, is vitally beneficial to the control of bacterial infections. It is also a major driver for aberrant inflammation in lethal sepsis [24]. Upon LPS, TLR4 rapidly assembles with MyD88 to activate down-stream signaling, contributing to the release of inflammatory genes [25]. By non-canonical pyroptosis, intracellular LPS activates pro-Caspase 11 in mice (Caspase 4/5 in humans), and it functions as an innate immune sensor by initiating an inflammatory response independent of TLR4. Besides Caspase 4/5/11, LPS directly activates Caspase 1 via binding to NLRP3, thereby potentiating canonical pyroptosis and generation of inflammatory cytokines. Furthermore, upon the stimulation of LPS, the complement system is activated through the alternative pathway, in which C3 is firstly activated and ultimately C5 is stimulated to deal with pathogens and provoke a series of inflammatory responses [26]. Despite that the intensive effect of complement components on inflammation has been largely accepted, the specific modulatory mechanism of C5a on septic inflammation remains to be clarified. Interestingly, C5a has been previously demonstrated to enhance inflammasome activity, thus mediating pyroptosis and promoting inflammatory response [27, 28].

The current study, for the first time, shed light on the relationship between C5a and pyroptosis in LPS-induced lung injury and confirmed the modulatory effects of C5a on cell pyroptosis. First, we demonstrated the protective effects of C5aR inhibition on LPS-induced lung injury both *in vivo* and *in vitro*. When C5aR was inhibited, the histological characteristics were ameliorated and inflammatory biomarkers were lowered in lung tissues and BALF, together with the ameliorative cell viability and inflammation in bronchial epithelial cell line. After detecting the effects of W-54011 on both apoptosis- and pyroptosis-related proteins, we observed obvious influence of W-54011 on the proteins involved in the pyroptosis pathway. TLR4 is known to activate NLRP3 via assembling with MyD88 upon LPS stimulation, thus activating Caspase 1 [29, 30]. These data unmasked that the actions of C5a/C5aR were mainly dependent on pyroptosis through TLR4 signaling. At the same time, W-54011 also exerted significant effect on HMGB1 expression. As a late inflammatory mediator, HMGB1 can be induced by LPS and it participates in pyroptosis during the pathogenesis of sepsis via the secretion of pro-inflammatory factors, such as TNF-α, IL-1, II-6, and IL-8 [31, 32]. The alteration of HMGB1 may be attributed to the notion that the blockade of pyroptosis by W-54011 prevented cell membrane from bursting and hindered the release of HMGB1, ultimately restricting the subsequent inflammatory response.

As for *in vitro* studies, the co-treatment of LPS and ATP was utilized to challenge cells because the ATP is the necessary energy carrier for Caspase 1 activation. In addition, we introduced C5a to treat cells, which illustrated that the activation effect of C5a on apoptosis and pyroptosis. Consistent with *in vivo* studies, results from cell model further confirmed our speculation that the actions of C5a/C5aR could be mainly dependent on pyroptosis pathway through TLR4 signaling.

## CONCLUSIONS

Taken together, the current study for the first time demonstrated the protective effect of C5aR inhibition on LPS-induced lung injury and uncovered the potential mechanism by inhibition of pyroptosis, thereby preventing inflammatory response. Therefore, our results not only enriched the understanding about the actions of complement system, but also provided novel evidence for the potential value of complement inhibitors in treating complement-dependent inflammatory diseases.

## MATERIALS AND METHODS

### Reagents

Lipopolysaccharide (LPS), W-54011 and ATP were purchased from MedChemExpress (MCE, USA). C5a was purchased from R&D Systems. Zoletil 50 was obtained from Vibac Laboratories (France).

### Animals and protocols

The Specific pathogen Free (SPF) adult male Sprague–Dawley (SD) rats were provided by the Beijing Vital River Laboratory Animal Technology Co., Ltd (Beijing, China). All procedures were performed following the Care and Use Guide of Laboratory Animals of the National Institutes of Health, with the approval of the Scientific Investigation Board of Shanghai Jiao Tong University, School of Medicine (SYXK-2018-0027). Forty rats were randomly assigned into four groups: Control, LPS, LPS+W-54011 1mg/kg and LPS+W-54011 5mg/kg. The induction of ALI was operated by intratracheal instillation of LPS as described previously [33, 34]. After anesthetizing via intraperitoneal (i.p.) injection of Zoletil 50 (10 mg/kg), the control animals were instilled intratracheally with 200 μL normal saline, while LPS-treated rats received 50 mg/kg LPS (dissolved in 200 μL saline; Sigma-Aldrich Co, USA) by intratracheal instillation. W-54011 (dissolved in 10% DMSO and then diluted using normal saline; i.p., q.d) was applied to pretreat rats 3 days before LPS instillation. To exclude the actions of DMSO, control and LPS groups also received the equal amount of DMSO. At 12 h after the LPS instillation, rats were anesthetized for next experiments.

### Cell culture and treatment

Human bronchial epithelial (Beas-2B) cells (American Type Culture Collection, ATCC) were cultured in 1640 medium (Abcam, UK) with 10% FBS (Wisent) and 1% antibiotics (Thermo Fisher Scientific, USA) in an atmosphere of 5% CO_2_ at 37° C. Cells were divided into five groups: Control, LPS+ATP, LPS+ATP+W-54011, LPS+ATP+C5a and LPS+ATP+C5a+W-54011. For C5a and W-54011 treatment, LPS (1 μg/ml) and ATP (5 mmol/L) were added to culture medium after pretreatment with W-54011 (10 μg/ml; dissolved in 10% DMSO and then diluted using PBS) and C5a (50 ng/ml) [35] for 12 h, separately. To exclude the actions of DMSO, control and LPS+ATP groups also treated with the equal amount of DMSO.

### CCK-8

For the assessment of cell viability, cells in the 96-well culture dishes after treatment were incubated with 10 μL of WST-8 working solution for 2 h under normal condition, and then the absorbance at 460nm was measured with a microplate reader.

### Histological staining

The left lung lobe was fixed with 4% paraformaldehyde, dehydrated and embedded in paraffin, and then cut into 5 μm thick section. After staining with hematoxylin and eosin (H&E) or Periodic Acid Schiff (PAS, Beyotime Biotechnology, China), lung tissue sections were observed with an optical microscope for pathological examination. Besides, some tissue sections were deparaffinized, rehydrated and subjected to TUNEL (Beyotime Institute of Biotechnology, Shanghai, China) staining, and the death of cells was observed under a fluorescence microscope.

### Detection of lung wet/dry ratio

After being sacrificed, the lungs of rats were isolated, and the weight of the lungs was immediately recorded as the wet weight. The blood on the lungs was blotted with filter paper, and the lungs were then stored in a 60° C incubator for 48 h. The weight of the lungs was recorded as dry weight. The lung wet/dry weight ratio (W/D) was used to assess the degree of pulmonary edema.

### Collection of broncho alveolar lavage fluid (BALF)

Before lavage, rats were anesthetized by injection of Zoletil 50 (10 mg/kg, i.p.). After exposing the chest cavity and intubating the trachea, 5mL PBS was slowly dropped into the lung bronchus 3 times for recovery. The collected solution was centrifuged at 1000 × g for 10 min at 4° C. The supernatant was collected and stored at -80° C for further analysis. The total protein concentration of BALF was determined by BCA method (Beyotime, China), and the sedimented cells were resuspended and subjected to cell counting using a hemocytometer.

### ELISA

The activities of inflammatory factors including IL-1β, IL-18, TNF-α, IL-6 and HMGB1 in BALF, lung tissues and cell supernatant, together with C5a levels in serum of rats, were detected by ELISA assay following the instructions (Abcam, Cambridge, UK) as described previously [33]. Results were expressed as optical density (OD) of 450 nm.

### Western blotting

The lung tissues were homogenized by the ultrasonication, and total proteins of lung tissues or BEAS-2B cells were extracted using RIPA lysis buffer (Thermo Fisher Scientific) supplemented with PMSF and cocktail (Roche, USA). After quantification, proteins were separated by sodium dodecyl sulfate polyacrylamide gel electrophoresis (SDS-PAGE) and transferred to PVDF membranes (Millipore, Billerica, MA, USA). After being blocked with 5% non-fat milk, the membranes were incubated with the following primary antibodies against Bax, Bcl-2, Caspase 1/3/4/5/11, HMGB1, toll like receptor 4 (TLR4), myeloid differentiation factor 88 (MyD88), and C5aR. The antibodies were further cultured with HRP-conjugated IgG (Abcam, UK) and visualized by chemiluminescence (Thermo Fisher Scientific). The overall gray value of the protein bands was quantified using Image J software (National Institutes of Health, USA).

### Flow cytometry

Cells were subjected to flow cytometry based on Annexin V/PI staining. Briefly, cells were washed with PBS and digested with 0.25% trypsin. Next, FITC-conjugated anti-annexin-V/PI staining antibody (BD Biosciences, USA) and propidium iodide solution were mixed and gently resuspended in Annexin V Binding buffer. After incubation with the cells, flow cytometry was performed using a flow cytometer (Becton Dickinson, USA). The data was analyzed by flow cytometry software.

### Statistical analysis

GraphPad Prism 6 software was used for statistical analysis and all data were expressed as mean ± SD. One-way ANOVA was used for mean comparison among groups. *P* value < 0.05 was considered statistically significant.
